# Non-structure protein ORF1ab (NSP8) in SARS-CoV-2 contains potential γδT cell epitopes

**DOI:** 10.3389/fmicb.2022.936272

**Published:** 2022-07-22

**Authors:** Boyu Du, Yang Guo, Gang Li, Yunhe Zhu, Yunfu Wang, Xueyan Xi

**Affiliations:** ^1^Institute of Basic Medical Science, Hubei University of Medicine, Shiyan, China; ^2^Hubei Key Laboratory of Embryonic Stem Cell Research, Hubei University of Medicine, Shiyan, China; ^3^Renmin Hospital, Hubei University of Medicine, Shiyan, China; ^4^Institute of Neuroscience, Hubei University of Medicine, Shiyan, China

**Keywords:** SARS-CoV-2, γδT cells, CDR3, ORF1ab, NSP8

## Abstract

Upon activation by the pathogen through T-cell receptors (TCRs), γδT cells suppress the pathogenic replication and thus play important roles against viral infections. Targeting SARS-CoV-2 *via* γδT cells provides alternative therapeutic strategies. However, little is known about the recognition of SARS-CoV-2 antigens by γδT cells. We discovered a specific Vγ9/δ2 CDR3 by analyzing γδT cells derived from the patients infected by SARS-CoV-2. Using a cell model exogenously expressing γδ-TCR established, we further screened the structural motifs within the CDR3 responsible for binding to γδ-TCR. Importantly, these sequences were mapped to NSP8, a non-structural protein in SARS-CoV-2. Our results suggest that NSP8 mediates the recognition by γδT cells and thus could serve as a potential target for vaccines.

## Introduction

Coronavirus disease 2019 (COVID-19) has been swept across the globe due to its extreme fast transmission speed and high pathogenic potential (Jin et al., 2020; Yang et al., 2020). By June 2022, there have been 529,410,287 confirmed cases of COVID-19, resulting in 6,296,771 deaths.^1^ Vaccination so far has been the key to the success in controlling the pandemic. With the danger of new variants looming around, more efforts are dedicated to developing alternative approaches for immunization.

γδT cells are increasingly recognized for important roles against viral infection (Rojas et al., 2002; Cao and He, 2005; Holtmeier and Kabelitz, 2005; Zhang et al., 2006). Primarily distributed within mucosa and subcutaneous tissues in skin, small intestine, lung, and reproductive organs, γδT cells account for 0.5–5% of peripheral blood mononuclear cells. Vγ9δ2T cells give rise to the main subtype of peripheral γδT cells. Virus-activated γδT cells could trigger a series of antiviral responses including release of cytokines (including IFN-γ, TNF-α, and IL-17), restriction of viral replication, and cytolysis of virus-infected cells (Jouan et al., 2020; Lei et al., 2020; Yazdanifar et al., 2020; Orumaa and Dunne, 2022). Multiple mechanisms have been proposed for the recognition of SARS-CoV-2 by γδT cells. TLRs (Toll-like receptors), a member of pattern recognition receptors, recognize SARS-CoV-2 RNA and mediate the activation of γδT cells (Hirsh and Junger, 2008). NKG2D receptors bind with MIAC/B and ULBP molecules that are expressed on the surface of SARS-CoV-2 infected cells (Ghadially et al., 2017). In addition, TCR receptor can bind with phosphorylated antigen and protein antigen (Spencer et al., 2008). In spite of phosphoantigen being regarded as the main γδ TCR-recognized antigen, phosphoantigen-activated γδT cells display restricted TCR diversity, and only a subset of phosphoantigen-responsive γδT cells mediate protective immunity against microorganisms (Rojas et al., 2002; Spencer et al., 2008; Morath and Schamel, 2020). Previously, we have observed that protein antigens could be recognized by γδT cells, and activated γδT cells could effectively induce innate and adaptive immunity against microorganisms (Boom et al., 1994; Xi et al., 2013, 2021). However, the entity of antigenic components in SARS-CoV-2 recognized by TCR remains obscure.

With a strategy for screening γδTCR-specific antigen epitopes established previously (Xi et al., 2011a,b, 2013), we revealed NSP8, a non-structural protein of SARS-CoV-2, as a strong candidate target for γδT cells mediated by γδTCR, thus opening up more space for the development of alternative vaccination schemes.

## Methods

### Subjects

Twenty COVID-19 patients were recruited at Xiyuan and Renmin Hospitals in Shiyan City, Hubei province, China. Ten healthy donors were recruited at Renmin Hospital in Shiyan City. The protocol for this study has received approval from the Clinical Ethics Committee of Hubei University of Medicine (No. 2020-TH-017). COVID-19 patients and healthy donors were all free from tumors, other infections, and diseases. All individuals had given their informed consent to participate in this study. The median ages of COVID-19 patients and healthy subjects were 42.8 and 39.3, respectively. The sex ratio for males and females is 12/8 in COVID-19 patients and 6/4 in healthy donors.

### RNA extraction and reverse transcription polymerase chain reaction

Total RNA was extracted separately from the peripheral blood of COVID-19 patients and healthy donors. One microgram of total RNA was then converted into cDNA using a reverse transcription system. Primer sequences complementary to upstream V regions and downstream C regions were used to amplify the CDR3 regions. The primer sequences were listed in Table 1.

**TABLE 1:** The primer sequences.

Primer name	Primer sequence
γ9CDR3-up	5′-AATGTAGAGAAACAGGAC-3′
γ9CDR3-down	5′-ATCTGTAATGATAAGCTTT-3′
δ2CDR3-up	5′-GCACCATCAGAGAGAGATGAAGGG-3′
δ2CDR3-down	5′-AAACGGATGGTTTGGTATGAGGC-3′
Sequencing primer 1	5′- TTATTCGCAATTCCTTTAGTG -3′
Sequencing primer 2	5′- GCCCTCATAGTTAGCGTAACG -3′

### Cloning and sequencing of Vγ9 and Vδ2 CDR3 regions

The purified PCR products were ligated into pGEM-T easy vector (Invitrogen, Carlsbad, CA, United States) and sequenced by using T7 primer (Sangon Biotech Inc., Shanghai, China). The CDR3γ region was considered to contain conserved “CALW” at its N-terminus and conserved “KVFG” at its C-terminus. While CDR3δ region was considered to contain conserved “CA” at its N-terminus and conserved “FGXG” at its C-terminus.

### Construction of SARS-CoV-2-specific γδTCR transfected cells

**FIGURE 1:**
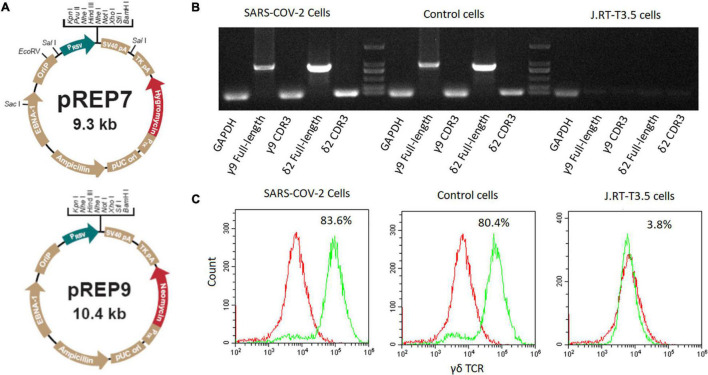
Construction of SARS-CoV-2 specific γδTCR transfected cells. **(A)** The map of pREP7 and pREP9 vectors. **(B)** Detection of γδTCR expression by PCR in transfected J.RT3-T3.5 cells. mRNA was extracted from transfected cells and reverse transcribed into cDNA. The full-length γ9 and δ2 chains and their CDR3 sequences were amplified by using specific PCR primers. **(C)** Detection of γδTCR expression by FACS analysis in J.RT3-T3.5 transfected cells. The cells were stained with FITC-labeled γδTCR antibody and then analyzed by flow cytometry on a MoFlo XDP flow cytometer. The results are representative of three independent experiments. SARS-CoV-2 cells: SARS-CoV-2 specific γδTCR transfected J.RT-T3.5 cells; Control cells: healthy controls’ γδTCR transfected J.RT-T3.5 cells. J.RT-T3.5 cells: J.RT-T3.5 cells without plasmid transfection.

The SARS-CoV-2 specific CDR3 sequences were separately inserted into full-length γ9 and δ2 chains to substitute their original CDR3 sequences based on our previous report (Xi et al., 2011b). The obtained γ9 and δ2 chains were then inserted into pREP7 and pREP9 vectors (Figure 1A), respectively. Full-length pREP7-γ9 and pREP9-δ2 chains were co-transfected into J.RT3-T3.5 cells. After 48 h, the transfected cells were cultured in a selection medium with hygromycin and neomycin for 4 weeks. The expression of transfected γδTCR in the cells was then evaluated by flow cytometry.

### *In vitro* panning

The transfected cells expressing potential SARS-CoV-2 specific γδTCR were used as probe cells to perform subtractive screening in a 12-peptide phage-display library. Four rounds of screening with conditions such as increased Tween concentration, increased action time with control cells as well as decreased action time with SARS-CoV-2 specific γδTCR transfected cells were conducted in order to enrich epitope peptides that could specifically bind with SARS-CoV-2 specific γδTCR transfected cells.

### Peptide synthesis

Sangon Biotech Inc. synthesized peptides with a purity of more than 95% as determined by high-performance liquid chromatography analysis. Half of the synthesized peptides were linked with FITC at their N-terminals.

### Flow cytometry

Cells were incubated with FITC-conjugated peptide or control peptide for 30 min at 4°C. The cells were then analyzed by flow cytometry on a MoFlo XDP flow cytometer (Beckman Coulter, Fullerton, CA, United States).

### Magnetic-activated cell sorting

γδT cells were isolated from healthy donors’ peripheral blood mononuclear cells (PBMCs) using an anti-TCR γ/δ MicroBead Kit from Miltenyi company (130-050-701) according to the manufacturer’s instructions.

### Protein-immobilized amplification assay

The transfected cells and sorted γδT cells were separately incubated with 10 ng/mL NSP8 protein (Sino Biological Inc., Beijing, China) or control protein for 30 min at room temperature. After extensive washing with RPMI-1640 culture medium, the transfected cells and natural γδT cells were then plated into 24-well plates at 1 × 10^6^ cells per well. The supernatants were harvested after 24 h and the level of IL-2 was detected by using Human IL-2 ELISA Kit (BD Biosciences, San Jose, CA, United States) according to the manufacture’s instructions.

### Bioinformatics analysis

The homologous analysis and sequence alignment were performed by using the Basic Local Alignment Search Tool (BLAST) to identify the matched proteins. After the screening, the obtained epitope peptide candidates were analyzed on the Heliquest website.^2^ The sequence alignment between peptide candidates and the downloaded SARS-CoV-2 ORF1ab sequence was performed by using DNAMAN8 software.

### Statistical analysis

Statistical comparisons between the experiment group and control group were performed by using the Student’s *t*-test. All data were analyzed either by SPSS 19.0 software or by GraphPad 8.0 software. *P* < 0.05 was considered statistically significant.

## Results

### A specific CDR3δ2 sequence derived from COVID-19 patients

The specificity in antigen recognition by TCR is primarily determined by the sequences within CDR3 region that are highly diverse. We isolated peripheral γδT cells from the patients infected by SARS-CoV-2 viruses and analyzed both Vγ9 CDR3 and Vδ2 TCR regions in comparison to the sequences derived from healthy donors. There was no significant variation in Vγ9 CDR3 region identified between the infected and control groups (Table 2). However, in Vδ2 region, we found a CDR3 sequence specifically present in most of the infected cases (Table 3).

**TABLE 2:** Deduced Vγ9 CDR3 amino acid sequences of COVID-19 patients and healthy donors^a^.

	Clone	V region	N/P region	J region	Frequency^b^
COVID-19 patients	1	CALWE	APQ	ELGKKIKVFG	8/60
	2	CALWE	VIS	ELGKKIKVFG	8/60
	3	CALWE	PPV	ELGKKIKVFG	3/60
	4	CALWE	VACY	ELGKKIKVFG	2/60
	5	CALWE	GIC	ELGKKIKVFG	2/60
	6	CALWE	KKA	ELGKKIKVFG	2/60
	7	CALWE	DEHK	ELGKKIKVFG	2/60
	8	CALWE	PYQ	ELGKKIKVFG	2/60
Healthy donors	1	*CALWE*	*VIS*	*ELGKKIKVFG*	*4/30*
	2	*CALWE*	*APG*	*ELGKKIKVFG*	*4/30*
	3	CALWE	SKR	ELGKKIKVFG	2/30
	4	CALWE	GETP	ELGKKIKVFG	1/30
	5	CALWE	PLAAA	ELGKKIKVFG	1/30
	6	CALWE	GNSY	ELGKKIKVFG	1/30
	7	CALW	RRSG	ELGKKIKVFG	1/30
	8	CALWE	QIIEF	ELGKKIKVFG	1/30

^a^Total RNA was extracted separately from the peripheral blood of COVID-19 patients and healthy donors. One microgram of total RNA was then converted into cDNA using a reverse transcription system. Primer sequences complementary to upstream V regions and downstream C regions were used to amplify the CDR3 regions. The purified PCR products were ligated into pGEM-T easy vector and sequenced. The CDR3γ region was considered to contain conserved “CALW” at its N-terminus and conserved “KVFG” at its C-terminus.

^b^Number of identical clones/total number of clones sequenced. Not all the sequencing results were listed in the table.

**TABLE 3:** Deduced Vδ2 CDR3 amino acid sequences of COVID-19 patients and healthy donors^a^.

	Clone	V region	N-D-N region	J region	Frequency^b^
COVID-19 patients	1	CACD	PLLGDASY	TDKLIFGKG	18/80
	2	CACD	VLGA	TDKLIFGKG	6/80
	3	CACD	RLSP	TDKLIFGKG	6/80
	4	CACD	TLVS	TDKLIFGKG	4/80
	5	CACD	VRLS	TDKLIFGKG	3/80
	6	CACD	SLLGDSEY	TDKLIFGKG	3/80
Healthy donors	1	CACD	RLGDTG	TDKLIFGKG	5/40
	2	CACD	*TLVS*	TDKLIFGKG	4/40
	3	CACD	PLEAP	TDKLIFGKG	3/40
	4	CACD	PLTS	TDKLIFGKG	2/40
	5	CACD	ALLI	TDKLIFGKG	2/40
	6	CACD	VLPG	TDKLIFGKG	2/40

^a^Total RNA was extracted separately from the peripheral blood of COVID-19 patients and healthy donors. One microgram of total RNA was then converted into cDNA using a reverse transcription system. Primer sequences complementary to upstream V regions and downstream C regions were used to amplify the CDR3 regions. The purified PCR products were ligated into pGEM-T easy vector and sequenced. The CDR3δ region was considered to contain conserved “CA” at its N-terminus and conserved “FGXG” at its C-terminus.

^b^Number of identical clones/total number of clones sequenced. Not all the sequencing results were listed in the table.

### The identification of SARS-CoV-2-specific γδTCRs binding epitopes

We amplified the sequences encoding γδTCRs derived from either infected patients or healthy individuals. The γ9 sequence (CALWEVISELGKKIKVFG) was identical between the two groups, whereas the δ2 sequences were different, with CACDPLLGDASYTDKLIFGKG from COVID-19 patients and CACDRLGDTGTDKLIFGKG from healthy individuals. We then established the expressions of full length γ9 and δ2 chains *via* introducing the designated vectors into J.RT3-T3.5 cells by electroporation (see more details in section “Materials and Methods”). After 4 weeks of selection with hygromycin and neomycin, the SARS-CoV-2-specific γδTCRs lines established were verified by both PCR (Figure 1B) and flow cytometry (Figure 1C).

Next, we used this cell model to screen potential epitopes recognized by this γδTCR based on a 12-mer random peptide phage-display library (E8110S, New England Biolabs, Hitchin, United Kingdom) (Xi et al., 2011b). There were 20 positive clones obtained as indicated by the phage-ELISA. Sequences derived from these clones were sequenced and gave rise to the dominant epitope candidates (SP1 to SP8) (Table 4).

**TABLE 4:** The sequence of epitope peptide candidates^a^.

Name	Sequence	Frequency^b^
SP1	KKLKKSLTLPLQ	6/20
SP2	YTPQLPSYAAFA	5/20
SP3	VSRHALWELQQS	4/20
SP4	SLNVAKSESCLH	1/20
SP5	YKVVIFDWRRSD	1/20
SP6	KDAHPESEFDRD	1/20
SP7	KHKHPPFDPSRP	1/20
SP8	AQTPVSYSPTTF	1/20

^a^According to the results of phage-ELISA, 20 phage clones that could specifically bind with SARS-CoV-2-specific γδTCR transfected cells were obtained and amplified by RT-PCR. The PCR products were then sequenced and the corresponding amino acid sequences were analyzed. Eight dominant epitope candidates (SP1 to SP8) were obtained.

^b^Number of identical clones/total number of clones sequenced.

### Identified dominant epitopes bind to SARS-CoV-2-specific γδTCR

**FIGURE 2:**
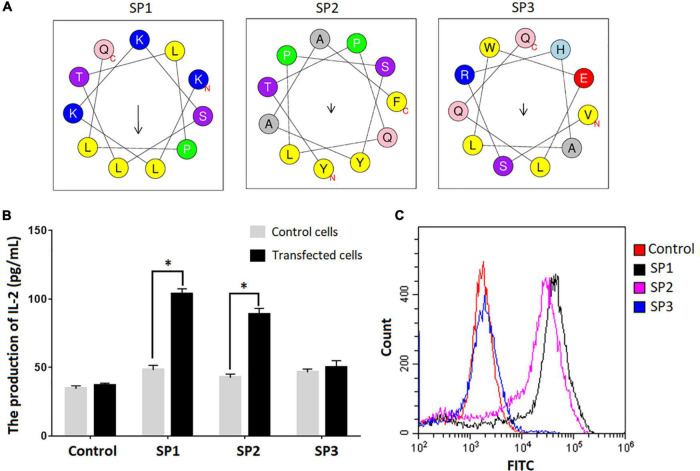
Confirmation of peptide binding to SARS-CoV-2 specific γδTCR transfected cells. **(A)** Spiral structure of three identified peptides predicted by bioinformatics tools on the Heliquest website (http://heliquest.ipmc.cnrs.fr/?tdsourcetag=s_pcqq_aiomsg). **(B)** IL-2 secretion after stimulation by the identified peptides in SARS-CoV-2 specific γδTCR transfected cells. The three peptides and control peptide were separately co-cultured with SARS-CoV-2 specific γδTCR transfected cells and control cells for 24 h. IL-2 production in the supernatant of the cell culture medium was detected by ELISA. Data was presented as mean ± SD from triplicate experiments. **(C)** The results of FACS analysis revealed the affinity between identified peptides and SARS-CoV-2 specific γδTCR transfected cells. The identified peptides and control peptides had been conjugated with FITC (10 μg) and were separately co-cultured with SARS-CoV-2-specific γδTCR transfected cells. The results showed that SP1 (76.3%) and SP2 (58.9%) could bind more effectively to the transfected cells than SP3 (2.78%). The results are representative of three independent experiments. ^∗^Denotes *p* < 0.05.

We used the SARS-CoV-2-specific γδTCR cell model to verify the binding between the epitopes identified and γδTCR. IL-2 secretion was monitored by ELISA in the cells upon the stimulation with individual epitope peptides. Among three representative epitopes SP1, SP2, and SP3 highlighted in Figure 2A (The predicted spiral structures were obtained using bioinformatics analysis tools proved by Heliquest website.), SP1 and SP2 triggered significant IL-2 production in the cells (Figure 2B) (*P* < 0.05). FACS analysis using FITC-conjugated peptides also confirmed that SP1 and SP2 exhibited strong affinity toward the SARS-CoV-2-specific γδTCR cells (Figure 2C).

### NSP8 protein in SARS-CoV-2 ORF1ab region contains potential epitopes that could activate γδT cells

**FIGURE 3:**
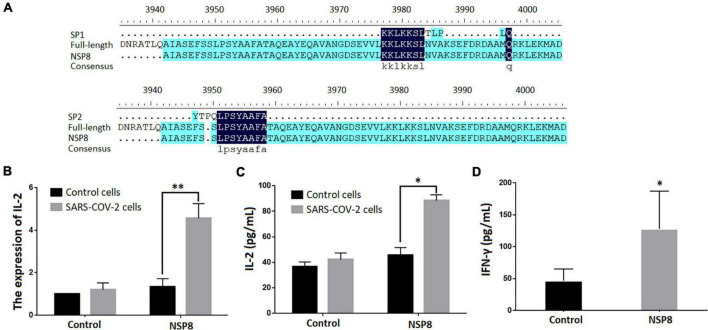
NSP8 protein contains potential epitopes that could activate γδT cell. **(A)** The sequence alignment of two peptide candidates with the sequence of SARS-CoV-2 and NSP8 protein sequence. **(B,C)** NSP8 protein could stimulate SARS-CoV-2 specific γδTCR transfected cells to produce more IL-2. The control protein and NSP8 protein were pre-coated in a 24-well plate. The SARS-CoV-2-specific γδTCR transfected cells were then added and cultured for 24 h. IL-2 secretion was measured either by RT-PCR (B) or by ELISA **(C)**. **(D)** NSP8 protein could bind to natural peripheral γδT cells. The control protein and NSP8 protein were pre-coated in a 24-well plate and incubated with γδT cells isolated from five healthy donors’ peripheral blood. IFN-γ secretion was measured in supernatants collected 24 h after incubation. Data were presented as mean ± SD from triplicate experiments. ^∗^Denotes *p* < 0.05; ^∗∗^Denotes *p* < 0.01.

A BLAST search was performed to identify SARS-CoV-2 proteins that contain SP1 and SP2 epitopes (Table 5). The top hits were located in ORF1ab region that encodes non-structural polyproteins involved in virus assembly, transcription, and replication. Further analysis using DNAMAN8 software revealed NSP8, among the ORF1ab region-derived polypeptides, as the origin of these γδTCR-specific epitopes (Figure 3A). NSP8 stimulates the production of IL-2 in the SARS-CoV-2-specific γδTCR cells, which were evident at both transcriptional (Real-time PCR, Figure 3B) and translational (ELISA, Figure 3C) levels. Furthermore, INF-γ production has been linked to γδT cell activation (Xi et al., 2011b,^2013^, ^2021^). Our findings also demonstrated that NSP8 could activate peripheral γδT cells isolated from healthy donors and increase INF-γ secretion in these cells, implying that NSP8 could bind to natural γδT cells (Figure 3D).

**TABLE 5:** BLAST analysis of epitope peptide candidates.

	Reference no.	Protein name	Species	E value	Matching part
SP1	UEX01438.1	ORF1a polyprotein	SARS-CoV-2	26.5	KKLKKSLT
SP1	UEX01439.1	ORF1ab polyprotein	SARS-CoV-2	26.5	KKLKKSLT
SP1	UMA92726.1	ORF1ab polyprotein	SARS-CoV-2	25.7	KKLKKSL L
SP2	UGC79169.1	ORF1ab polyprotein	SARS-CoV-2	27.8	LPSYAAFA
SP2	UJY79755.1	ORF1ab polyprotein	SARS-CoV-2	27.8	LPSYAAFA
SP2	UJE21816.1	ORF1ab polyprotein	SARS-CoV-2	27.8	LPSYAAFA

## Discussion

Similar to αβT cells, γδT cells secrete granzyme and perforin that target infected cells. This action normally is in conjunction with the expressions of FasL and TNF related apoptosis inducing ligand (TRAIL) that render targeted cells for apoptosis. In parallel, γδT cells orchestrate other immune cells to participate in antiviral responses, which is mainly mediated by cytokines and membrane molecules derived from γδT cells (Holtmeier and Kabelitz, 2005; Zhang et al., 2006; Carissimo et al., 2020; Lo Presti et al., 2021). However, unlike the case of αβT subtype, the recognition of antigens by γδT cells does not require antigen presentation from antigen-presenting cells (APC) (Cao and He, 2005; Xi et al., 2009, 2010), thus making this population of T cells attractive for alternative anti-infectious therapeutic development (Odak et al., 2020; Rijkers et al., 2020). We previously established a γδTCR *ex vivo* expression cell model for identifying antigens recognizable by γδT cells (Xi et al., 2011a,b, 2013). Here we took this approach to identify potential antigens of SARS-CoV-2 specific for γδT cells. SP1 and SP2 peptides identified from the screen exhibited strong affinity toward SARS-CoV-2-specific γδTCRs. Interestingly, it appeared that SARS-CoV-2 ORF1ab regions harbor the sequences encoding these epitopes. Specifically, NSP8, a non-structural protein, contains the sequences matching both epitopes. Considering the limitations within our screen model, further study is needed to test the effects of NSP8 protein on COVID-19 patients’ peripheral γδT cells.

The polyprotein encoded by ORF1ab gene segment is composed of sixteen non-structural proteins including NSP8 (Biswas et al., 2021). NSP8 initiates the synthesis of complementary short oligonucleotides and provides RNA primers required by NSP12 during viral replication and transcription (Imbert et al., 2006). It has been suggested that NSP8, being engaged in specific cytoplasmic foci, can form complexes with NSP7, NSP9, and NSP10 (Zhai et al., 2005; Achour, 2021) and suppress protein integration into cytoplasmic membrane thereby mitigate the interferon response of host cells (Banerjee et al., 2020; Gu et al., 2022). Recent studies highlighted the possibility of NSP8 as an antigenic target of SARS-CoV-2 (Ahmad et al., 2020; Ong et al., 2020). Our results reveal that NSP8 mediates the recognition of SARS-CoV-2 by γδTCR (Figure 3). This finding provides new opportunities for developing alternative vaccines through targeting non-structural proteins, which is also encouraged by the study of another non-structural protein, NS1, showing interesting potentials in both promoting immune protection and reducing viral replication (Salat et al., 2020). Moreover, antibodies induced by non-structural protein vaccines can bypass the issue with antibody-dependent enhancement (ADE).

SARS-CoV-2 variants can evade vaccine-induced immunity, leading to increases in transmissibility, infectivity, hospitalization, and mortality (Alkhatib et al., 2021; Singh et al., 2021). Importantly, we did not detect any hotspots of mutation related to all variants identified so far in SP1 and SP2 epitopes (data not shown). Few genomic alterations occur in the NSP8-encoding sequences (Koyama et al., 2020), highly likely due to the fact that no significant positive selection pressure upon these sequences as indicated by the *in silico* analysis^3^ (data not shown).

## Data availability statement

The original contributions presented in this study are included in the article/supplementary material, further inquiries can be directed to the corresponding authors.

## Ethics statement

The studies involving human participants were reviewed and approved by the protocol for our study had received approvals from the Clinical Ethics Committee of Hubei University of Medicine, Shiyan City (No. 2020-TH-017). The patients/participants provided their written informed consent to participate in this study.

## Author contributions

XX and YW conceived and designed the experiments. BD and YG performed the experiments. XX, BD, and YW analyzed the data. YZ and GL contributed to the reagents, materials, and/or analysis tools. XX and BD wrote the manuscript. All authors contributed to the article and approved the submitted version.

## Conflict of interest

The authors declare that the research was conducted in the absence of any commercial or financial relationships that could be construed as a potential conflict of interest.

## Publisher’s note

All claims expressed in this article are solely those of the authors and do not necessarily represent those of their affiliated organizations, or those of the publisher, the editors and the reviewers. Any product that may be evaluated in this article, or claim that may be made by its manufacturer, is not guaranteed or endorsed by the publisher.

## Footnotes

1
https://covid19.who.int/

2
http://heliquest.ipmc.cnrs.fr

3
http://www.datamonkey.org/meme
